# Analysis of factors affecting the clinical management of infection in culture-negative patients following percutaneous endoscopic decompression: a retrospective study

**DOI:** 10.3389/fcimb.2025.1540970

**Published:** 2025-01-27

**Authors:** Changpeng Qu, Haixin Wei, Hao Zhang, Zheng Lian, Hui Lu, Shuo Han

**Affiliations:** Department of Spine Surgery, The Affiliated Hospital of Qingdao University, Qingdao, China

**Keywords:** spinal surgery, postoperative infection, BMI, endoscope, percutaneous endoscopic decompression, culture-negative

## Abstract

**Background:**

Culture-negative spinal infections after prophylactic antibiotic use in percutaneous endoscopic decompression are rare, and diagnostic difficulties and the risk of antibiotic-resistant infections complicate treatment. This study retrospectively analyzed the medical records of culture-negative patients following percutaneous endoscopic surgery to identify risk factors influencing antimicrobial therapy and provide insights for clinical management.

**Methods:**

Data were retrospectively collected from patients who underwent lumbar percutaneous endoscopic decompression at the Affiliated Hospital of Qingdao University between January 2014 and June 2023. The patients’ medical records were reviewed. Patient demographics, hidden blood loss, daily blood glucose control, and maximum temperature during treatment were recorded as potential risk factors. C-reactive protein, procalcitonin, white blood cells, erythrocyte sedimentation rate, and the duration of antibiotic treatment were used as indicators of infection treatment. The impact of these risk factors on infection was then analyzed.

**Results:**

The results showed that blood glucose control was strongly correlated with the severity of infection (Beta = 0.60, P = 0.00), strongly correlated with short-term treatment effectiveness (Beta = 0.65, P = 0.00), and moderately correlated with the duration of antibiotic treatment (Beta = 0.41, P = 0.01). Hidden blood loss was moderately correlated with the severity of infection (Partial-R = 0.49, P = 0.00) and moderately correlated with the duration of antibiotic treatment (Partial-R = 0.48, P = 0.00). Hidden blood loss index was moderately correlated with the duration of antibiotic treatment (Partial-R = 0.50, P = 0.00). Female was a favorable factor to shorten the duration of antibiotic treatment (Beta = -0.25, P = 0.01), and higher maximum temperature during infection may indicate a longer duration of antibiotic treatment (Beta = 0.28, P = 0.02).

**Conclusion:**

Our findings suggest that healthy blood glucose levels, a lower hidden blood loss and hidden blood loss index might help reduce the duration of antibiotic use after infection. Effective hemostasis during surgery to reduce hidden blood loss and good preoperative blood glucose control indicators are both beneficial measures for infection treatment.

## 1 Introduction

Lumbar disc herniation (LDH) and lumbar spinal stenosis (LSS) are the most common degenerative diseases of the lumbar spine (Katz et al., 2022; Souslian and Patel, 2024). Due to structural changes, surgical intervention is often necessary for treatment. As surgical techniques and concepts have advanced, the approach to treating lumbar degenerative diseases has evolved from posterior lumbar interbody fusion (PLIF) to percutaneous endoscopic decompression (PED) (Lee et al., 2011; Elkatatny et al., 2019). Compared to PLIF, PED offers several advantages, including shorter operation time, less trauma, faster recovery, and lower complication rates (Sidhu et al., 2014). Previous studies have shown that PED is less invasive than PLIF and has a lower incidence of surgical site infections (Miscusi et al., 2023), while achieving similar treatment outcomes. Some studies have indicated that operation time is a key risk factor for SSI, and that the use of percutaneous endoscopic techniques is a significant protective factor in preventing SSI, and currently, there is no evidence that different degenerative lumbar spine diseases affect the incidence of SSI (Wang et al., 2014; Lee et al., 2016). Additionally, large prospective studies have found that the likelihood of developing SSI after PED is approximately one-third of that following PLIF (Ogihara et al., 2018).

**Figure 1:**
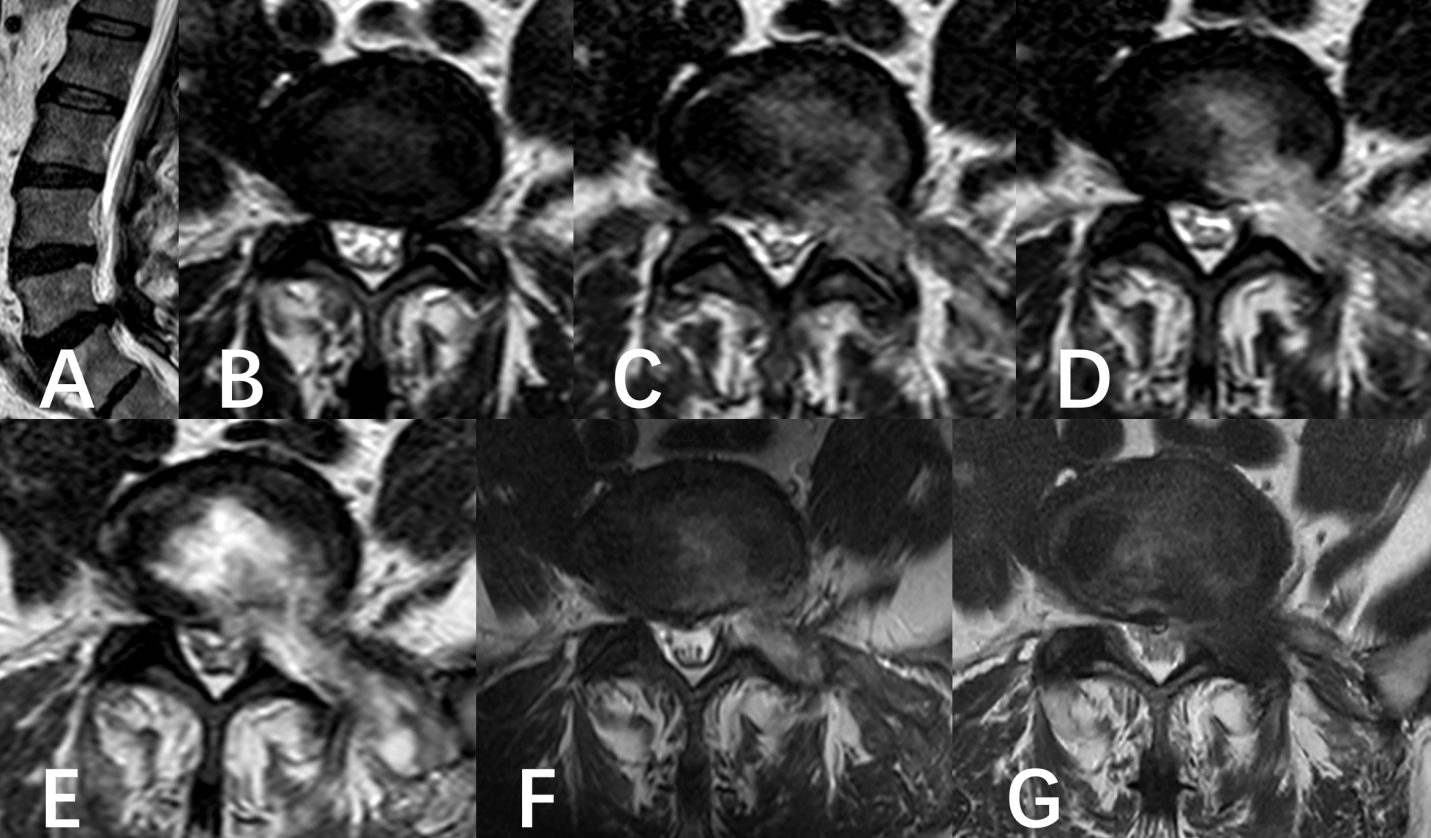
A six-year-old male was diagnosed with L5/S1 lumbar disc herniation and underwent PED. **(A, B)** Preoperative MR showed the left disc herniation before operation. **(C–F)** Follow-up of postoperative MRI images. **(C)** showed the state of the operation area immediately after operation. **(D)** was the state of operation area at the beginning of anti-infection treatment. **(E)** was the state of the operating area near the most serious period of infection captured in the anti-infection process. **(F)** was the result of the last follow-up before discharge. **(G)** was the result of 6 months follow-up.

Although the postoperative infection rate of PED is lower than that of PLIF, postoperative infection after PED cannot be completely avoided. What’s worse, routine preoperative antibiotic use makes it more likely to obtain negative bacterial cultures when SSI occurs after PED. Even if bacterial culture results are negative, symptoms such as changes in the patient’s condition, elevated infection markers, and imaging findings indicating tissue edema at the surgical site all suggest the presence of postoperative infection (Dai et al., 2020). Due to the use of preoperative prophylactic antibiotics, the likelihood of antibiotic-resistant bacteria emerging in postoperative SSI increases (McCullough et al., 2016; Macki et al., 2022). Some studies have found that patients with culture-negative have lower infection markers, while a higher body temperature (>37.8°C) may be a favorable factor for positive culture results (Yu et al., 2020). In common pyogenic spinal infections, spinal surgeons often rely on experience to administer broad-spectrum antibiotics and determine whether the patient has an SSI, based on the effectiveness of antibiotic treatment and changes in laboratory indicators, as it is difficult to find other serological tests available to help identify the infecting pathogen (Tali, 2004). If the patient’s symptoms improve, infection markers decrease, body temperature normalizes, and imaging findings show gradual improvement after antibiotic treatment, this generally suggests that the infection diagnosis was correct (**Figure 1**). Conversely, it may indicate a transient inflammatory response due to aseptic inflammation. At this stage, the physician loses the ability to actively target the infection and must passively accept the treatment outcomes before deciding on the next course of action. In terms of treatment prognosis and effectiveness, after the appropriate use of antibiotics, there is no difference between patients with negative cultures and those with positive cultures (Dai et al., 2020). However, an antibiotic use duration shorter than 6-8 weeks may be a high-risk factor for infection recurrence (McHenry et al., 2002; Kim et al., 2014). The risk of bacterial resistance associated with long-term antibiotic use increases accordingly. With the increasing risk of antibiotic resistance and the interference of negative bacterial cultures in diagnosis, the infection may progress silently, leading to a sudden worsening of clinical symptoms, and potentially resulting in shock or death (Kim et al., 2015; Santoso et al., 2018; Rabin et al., 2023). Therefore, when empirically using antibiotics in postoperative SSI patients following PED, shortening the treatment duration while controlling infection progression is a favorable factor in preventing adverse outcomes.

In assessing the progression of infections, inflammatory markers serve as an important reference for clinical judgment. Previous studies have shown that patients with negative cultures may have lower inflammatory markers, and approximately 50% of infections caused by gram-negative bacteria or fungi can present with normal C-reactive protein (CRP) levels. Research has also demonstrated that the impact of different types of bacterial infections on inflammatory markers varies, which provides some basis for empirical treatment decisions. Furthermore, even in the absence of overt symptoms or elevated inflammatory markers, if there is strong suspicion based on radiological findings, it is recommended to administer prolonged, adequate anti-infective therapy (Ghassibi et al., 2021). Specifically, in patients with postoperative spinal infections, some studies indicate that CRP may have higher sensitivity. Procalcitonin (PCT) can be mildly elevated in localized lumbar infections and significantly raised in systemic infections. PCT can be used as a reference marker for monitoring infection progression and treatment effectiveness (Jeong et al., 2015). However, since postoperative SSI of the spine are often localized, the diagnostic value of CRP is higher than that of PCT in these cases. The erythrocyte sedimentation rate (ESR) has high sensitivity for diagnosing SSIs, but due to its low specificity, it is less useful for assessing the effectiveness of anti-infective treatment. White blood cell counts (WBCs) can serve as another indicator for determining the presence of infection (Zhang et al., 2016).

Previous studies have mostly focused on identifying the risk factors for infections following spinal endoscopic surgery. Previous studies have suggested that factors such as advanced age (Klein et al., 1996), male gender (Rao et al., 2011), obesity (Wimmer et al., 1998), and diabetes (Wimmer et al., 1998) are risk factors for SSI. Through our research, we hoped to identify the factors that influence the progression and severity of infections in patients with culture-negative infections during actual clinical treatment. By intervening in these factors, we aimed to prevent the hidden development of severe infections, thereby ensuring that our clinical anti-infection treatments remain stable and controllable.

## 2 Materials and methods

### 2.1 Patient population

We conducted a review of the medical records of patients who underwent PED at the Affiliated Hospital of Qingdao University between January 2014 and June 2023, utilizing the Hospital Information System (HIS). In this study, SSI was defined according to the Centers for Disease Control and Prevention criteria (Mangram et al., 1999). A superficial SSI is defined as an infection that affects only the skin or subcutaneous tissue and occurs within 30 days post-surgery. A deep SSI is characterized as an infection that occurs within 30 days of the surgery (if no implant was used) or within one year (if an implant was present). This infection is considered surgery-related and involves deep soft tissues. A deep SSI is further classified by the presence of one or more of the following criteria (1): Purulent drainage from the deep incision (2); A deep incision that spontaneously dehisces or is intentionally opened by the surgeon when the patient exhibits at least one of the following symptoms: fever (>38.0°C), localized pain, or tenderness, unless the site is culture-negative (3); An abscess or other signs of infection involving the deep incision, identified via direct examination, reoperation, or histopathologic or radiologic findings (4); A diagnosis of deep incisional SSI made by the surgeon or attending physician (Mangram et al., 1999). Based on these criteria, we established the inclusion and exclusion criteria for this study as follows:

Inclusion criteria (1): PED treatment was performed due to LDH and/or LSS. Ineffective conservative treatment for more than 6 months, requiring single-segment surgical intervention (2); Age > 18 years (Lee et al., 2011); Preoperative prophylactic administration of 2g ceftriaxone via intravenous infusion, administered 2 hours before surgery (4); Three days after the operation, increase in body temperature(T>38.0°C), imaging findings suggestive of infection, laboratory test results indicate elevated infection markers (5); The results of repeated blood culture and tissue culture were negative.

Exclusion criteria (1): History of previous lumbar spine surgery (2); Pre-existing lumbar spine tumors, lumbar instability, or lumbar infections prior to PED; (3) Autoimmune diseases or long-term use of glucocorticoid; (4) Severe osteoporosis and/or fractures; (5) Incomplete key information.

Based on the inclusion and exclusion criteria, a total of 57 patients who developed infections with negative bacterial cultures after PED surgery were selected for this study. This was a retrospective study approved by the Medical Ethics Committee of the Affiliated Hospital of Qingdao University. Participants were not required to provide additional written informed consent.

### 2.2 Demographic and perioperative data collection

This study retrospectively collected patient demographic data, surgical-related information, laboratory indicators, visual analogue scale (VAS) scores for pain, and imaging data related to the surgical site through the HIS. The included demographic data consisted of age, sex, height, weight, body mass index(BMI), preoperative blood glucose control (BGC), maximum temperature (MT) during the infection period, preoperative blood volume (PBV), total blood loss (TBL), volume of irrigation fluid used during endoscopic surgery, red blood cell (RBC) count in the irrigation fluid sample, surgery duration, visible blood loss (VBL), hidden blood loss (HBL), HBL index (HBLI), and duration of antibiotic treatment (DAT).

Laboratory indicators included preoperative RBC count, preoperative hematocrit (Hctpre), postoperative hematocrit (Hctpost), and the levels of CRP, PCT, ESR, and WBCs. CRP, PCT, ESR and WBCs were recorded at four time points: at time point 1(T1)-the start of anti-infection treatment, at T2-the peak value, at T3- subsequent test near the peak (approximately 3-5 days after the peak), and at T4-the last inspection before discharge. VAS scores were also recorded at these four time points.

Based on the literature review, we defined age, sex, BMI, HBL, MT during treatment, and BGC as risk factors. Additionally, to account for individual differences related to preoperative blood volume, height, and weight, we developed the HBLI, calculated using the following formula: HBLI = HBL/PBV. HBLI was risk factor, too. We defined CRP, PCT, WBCs, ESR, VAS scores, and the DAT as indicators for assessing the severity of infection and treatment progress status.

### 2.3 Perioperative patient management

All surgeries were performed under general anesthesia by the same surgical team. The choice between percutaneous endoscopic interlaminar decompression (PEID) and percutaneous endoscopic transforaminal decompression (PETD) was made flexibly based on the patient’s specific condition. All patients received a prophylactic intravenous infusion of 2g ceftriaxone mixed with 100ml normal saline prior to surgery. According to “Expert Consensus on Perioperative Fluid Therapy for Surgical Patients (China, 2015),” to maintain a stable fluid balance, the total amount of intravenous fluids administered on the day of surgery was calculated at: 30mL/(kg·d) (Simpson et al., 2017). Preoperative and postoperative blood routine tests were performed on the morning of the surgery and in the evening after the surgery, both on an empty stomach.

### 2.4 Calculation formula

PBV was calculated according to the formula of Nadler.: PBV=k1×height(m^3^) + k2×weight (kg) + k3 (for male: k1 = 0.3669, k2 = 0.03219, and k3 = 0.6041; for female: k1 = 0.3561, k2 = 0.03308, and k3 = 0.1833) (Nadler et al., 1962).

TBL was calculated by multiplying PBV by the change of Hct according to the Gross formula (Gross, 1983; Hu et al., 2022): TBL=PBV (Hctpre−Hctpost)/Hctave, Hctave is the average of Hctpre and Hctpost. VBL = RBC of irrigation fluid sample ×10000 ×dilution multiple×K×total volume of irrigation fluid ÷ (preoperative RBC count×10^9^). Finally, HBL was calculated according to the formula of Sehat et al: HBL=TBL−VBL (Pattison et al., 1973).

**Figure 2:**
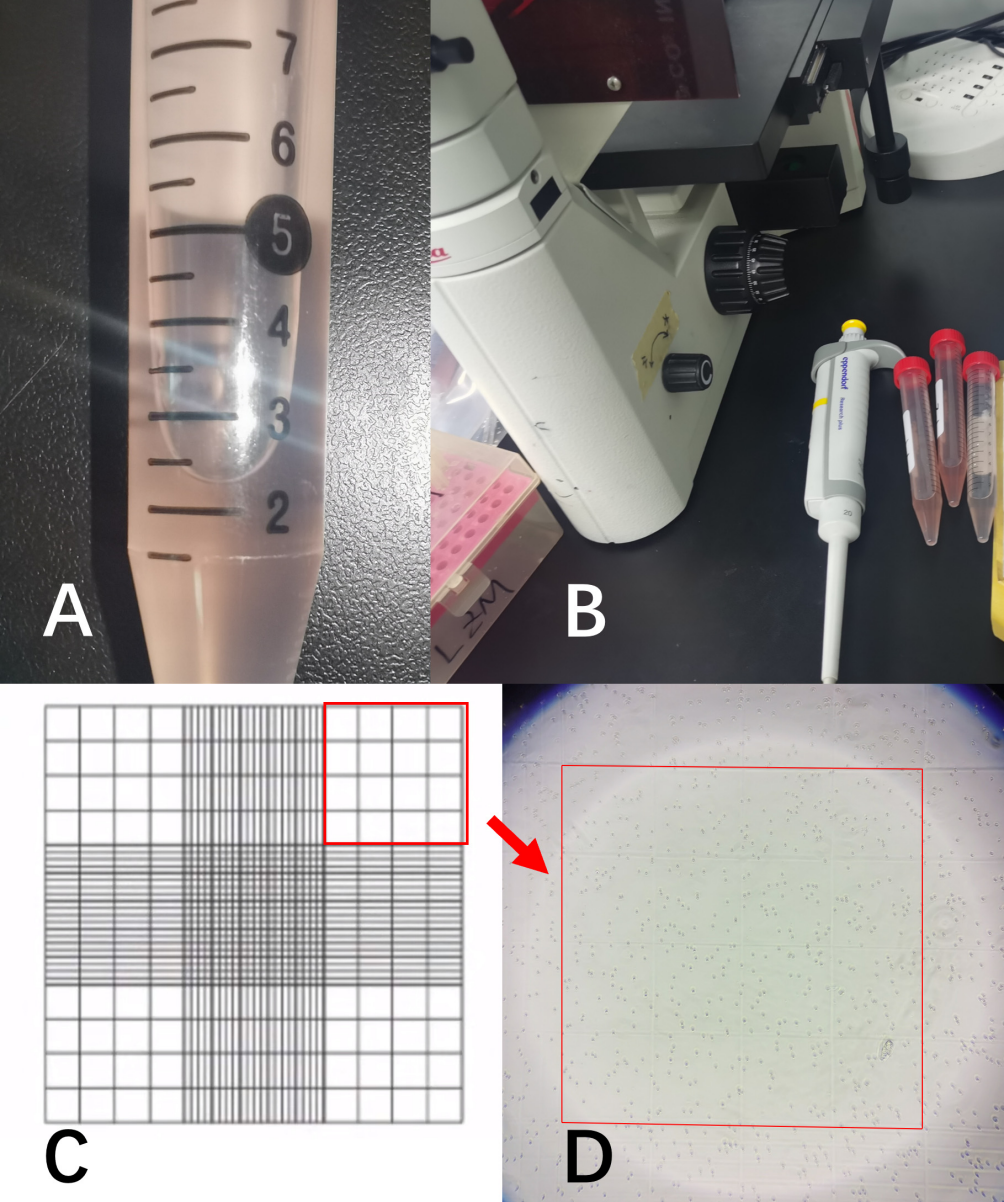
**(A)** represented the irrigation fluid sample; **(B)** showed the microscope and micropipette used for cell counting; **(C)** was a schematic diagram of the cell counting chamber. All samples were diluted to the appropriate ratio and counted using the 4×4 grid in the upper-right corner; **(D)** showed the RBCs in the microscope field of view.

After thoroughly mixing the irrigation fluid, a micropipette was used to withdraw a sample, which was then counted under a microscope using a hemocytometer (**Figure 2**). The dilution multiple and the constant K were assigned values (where K=1,16, or 25) according to actual counting methods and situation.

### 2.5 Statistical analysis

Statistical analysis was conducted using SPSS 26.0 (IBM, USA). Continuous data were presented as the means ± standard deviations. The normality of continuous variables was assessed using the Shapiro−Wilk test. To avoid errors caused by repeated measurements, repeated measures ANOVA was used to compare the differences in CRP, PCT, ESR, WBCs, and VAS across four time points (T1, T2, T3, and T4). Mauchly’s Test was performed to assess the sphericity assumption, and when the assumption was violated, the Greenhouse-Geisser correction was applied to adjust the degrees of freedom. Bonferroni correction was used to control the false positive rate for multiple comparisons. For correlation screening, Spearman’s rank correlation test or Pearson’s correlation test was employed (0.8-1.0: very strong correlation, 0.6-0.8: strong correlation, 0.4-0.6: moderate correlation, 0.2-0.4: weak correlation, 0.0-0.2: very weak or no correlation.). Select indicators that can reflect the dynamics of treatment for multiple linear regression analysis (correlated at T2 and/or DAT-related, R > 0.2 and P < 0.05, with correlation weakening as treatment progresses). For categorical variables, they were transformed into dummy variables. For continuous variables, the standardized regression coefficients and their confidence intervals were calculated to reflect the actual impact on the dependent variable, and the standardized regression coefficients were used to explore the relative weights of the independent variables. To avoid multicollinearity in the multiple regression model, collinearity diagnostics were performed on all independent variables, and the variance inflation factor (VIF) was calculated. A VIF > 10 indicated strong multicollinearity between variables, and those variables were excluded from the multiple linear model for partial correlation analysis. The correlation heat map was drawn using ChiPlot (https://www.chiplot.online/). A P value of less than 0.05 was considered statistically significant.

## 3 Results

**Figure 3:**
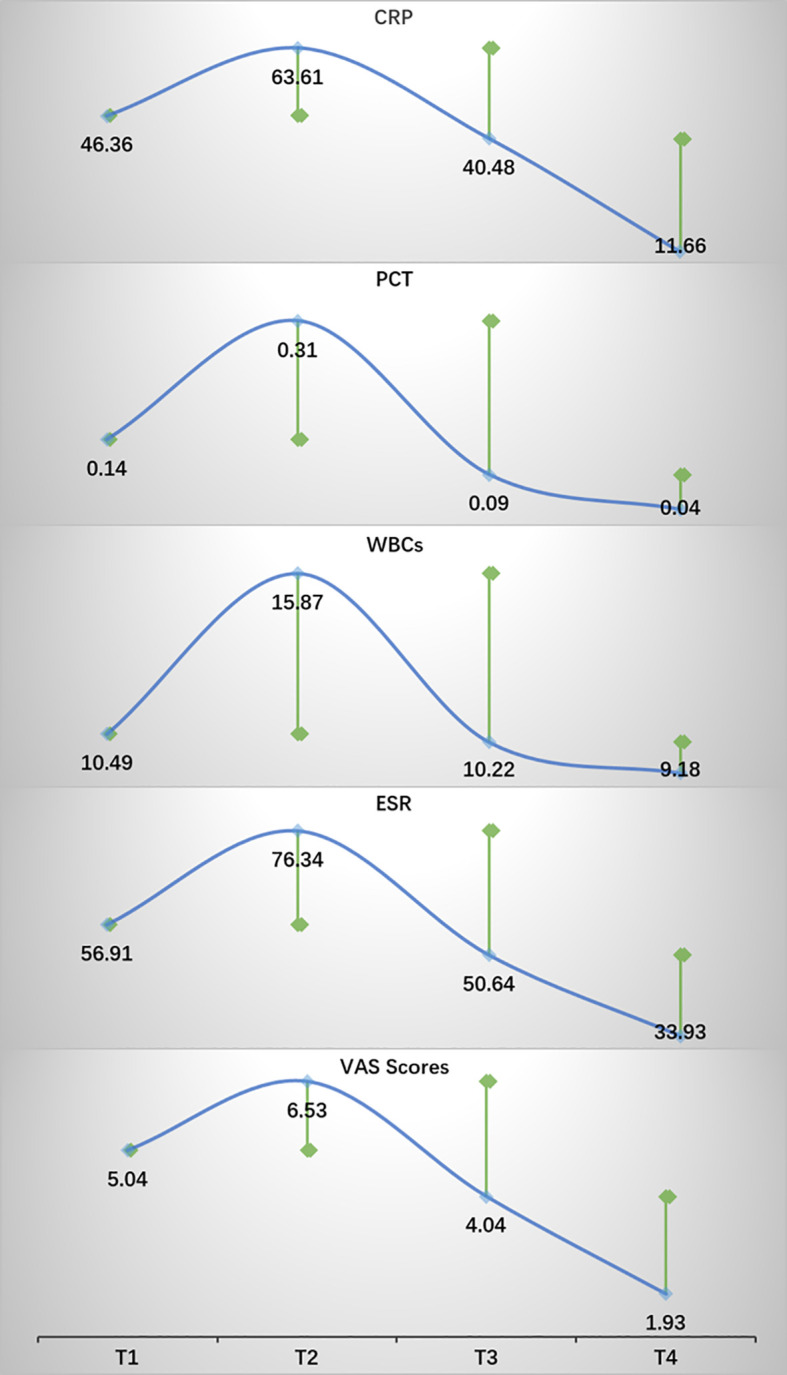
The trends in changes of CRP, PCT, ESR, WBCs, and VAS scores. Green square points represented statistical differences compared with the previous time point. P < 0.05.

After conducting a search through the HIS, a total of 57 patients met the inclusion and exclusion criteria for this study. Normality tests revealed that the continuous variables followed a normal distribution. The study included 29 males and 28 females, with an average age of 59.72 ± 9.76 years, an average height of 163.86 ± 7.20 cm, an average weight of 67.68 ± 8.66 kg, and a BMI of 25.21 ± 2.78kg/m^2^. Detailed information was provided in **Table 1**. The results of statistical data of bleeding volume in endoscopic surgery were shown in **Table 2**. The DAT, VAS scores, laboratory indicators of CRP, PCT, ESR, and WBCs were shown in **Table 3**. The changes in CRP, PCT, ESR, WBCs, and VAS scores are shown in **Figure 3**. The results indicated that the four time points showed statistically significant differences and could represent the severity of infection and treatment effectiveness at four different stages.

**Table 1:** Baseline data of participants.

Variable	Statistic
Number of patients	57
Sex	
Males	28
Females	29
Age (years)	59.72 ± 9.76
Height (cm)	163.86 ± 7.20
Weight (kg)	67.68 ± 8.66
BMI(kg/m^2^)	25.21 ± 2.78
BGC	
Level 1 (<7.0mmol/L)	14
Level 2 (7.0-11.0mmol/L)	21
Level 3 (>11.0mmol/L)	22
MT (°C)	38.29 ± 0.34

**Table 2:** Data of bleeding volume in endoscopic surgery.

Variable	Statistic
Preoperative RBC count (10^12/L)	4.71 ± 0.46
Hctpre (%)	42.73 ± 3.31
Hctpost (%)	38.55 ± 3.02
PBV (ml)	4278.02 ± 603.52
TBL (ml)	438.89 ± 138.11
Irrigation fluid volume (ml)	13157.89 ± 2396.35
Surgical duration (minute)	122.02 ± 26.24
RBCs of irrigation fluid sample	630.35 ± 160.97
VBL (ml)	17.67 ± 5.43
HBL (ml)	421.22 ± 137.69
HBLI	0.10 ± 0.03

**Table 3:** DAT, VAS scores, laboratory indicators of CRP, PCT, ESR, and WBCs.

Variable	Statistic
CRP (mg/L)	
T1	46.36 ± 23.12
T2	63.61 ± 21.15
T3	40.48 ± 24.97
T4	11.66 ± 12.68
PCT (ng/ml)	
T1	0.14 ± 0.11
T2	0.31 ± 0.16
T3	0.09 ± 0.10
T4	0.04 ± 0.03
ESR (mm/h)	
T1	56.91 ± 25.22
T2	76.34 ± 28.32
T3	50.64 ± 27.31
T4	33.93 ± 22.88
WBCs (10^9/L)	
T1	10.49 ± 2.80
T2	15.87 ± 3.10
T3	10.22 ± 2.52
T4	9.18 ± 2.59
VAS	
T1	5.04 ± 1.88
T2	6.53 ± 1.97
T3	4.04 ± 1.45
T4	1.93 ± 1.67
DAT (days)	28.04 ± 9.42

**Figure 4:**
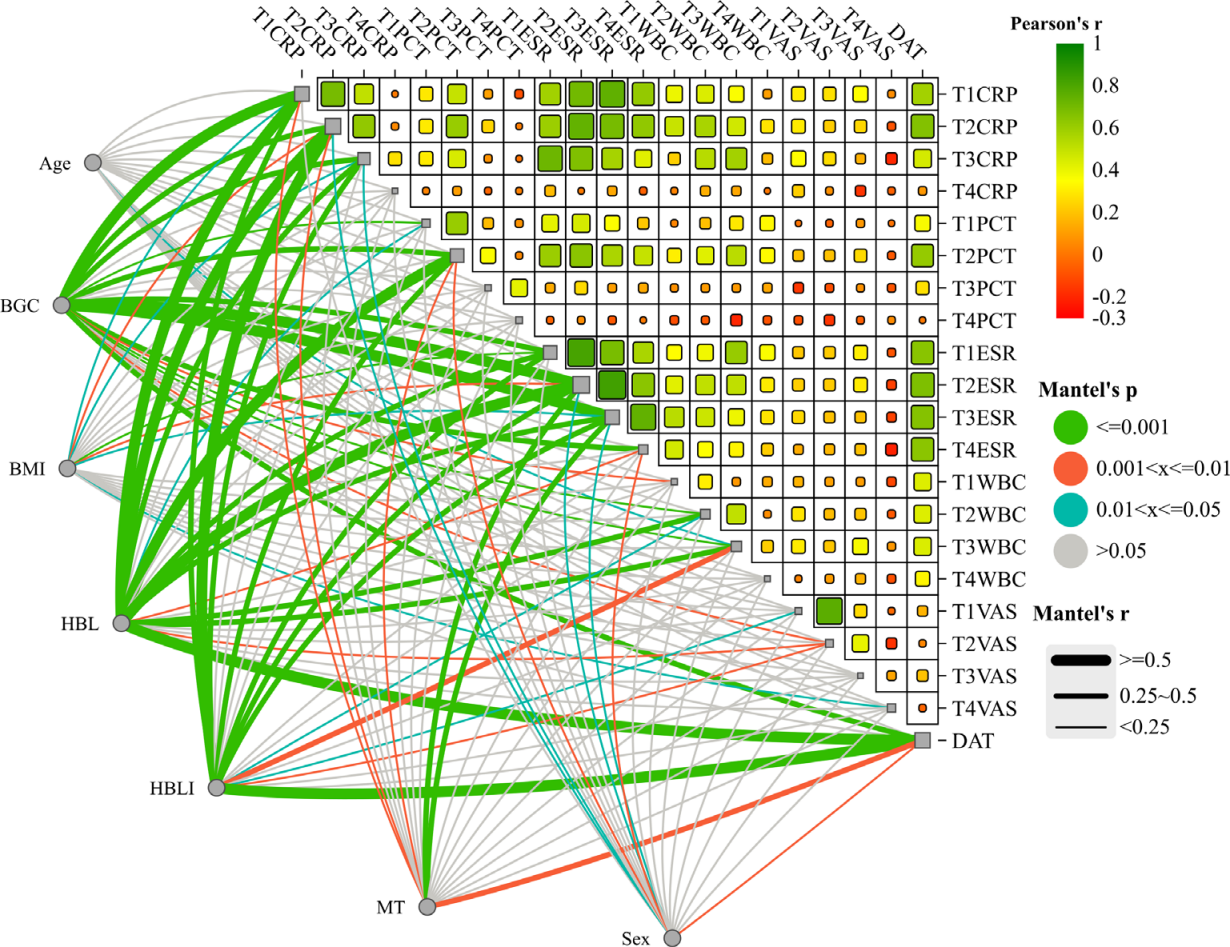
The upper-right section of the image shows the heatmap of the correlation analysis within the treatment effect and/or infection severity groups. Red represents positive correlation, and green represents negative correlation. This study did not explore this in depth and presents it as an additional statistical analysis result for the readers’ reference. The lower-left section of the image shows the interactive Mantel test correlation heatmap. The thickness of the lines represents the strength of the correlation, and the color of the lines indicates the P-value of the correlation.

In the correlation screening, sex, BMI, MT, BGC, HBL, and HBLI were all found to be correlated with DAT, and P < 0.05. Meanwhile, CRP at T2 and T3, PCT at T2, ESR from T1 to T4, and WBCs at T2 all showed good correlations with some of the independent variables. The specific results were shown in **Table 4**. The visualization results were shown in **Figure 4**. Since CRP has a high diagnostic value for spinal infections and can reflect the effectiveness of short-term medication, and the highest value of PCT can reflect the severity and progression of infection, while DAT directly reflects the duration of the treatment, CRP at T2 and T3 time points, PCT at T2 time point, and DAT were selected as dependent variables. T2PCT represents the severity of infection, T3CRP reflects the effect of short-term antibiotic use, and DAT represents the duration of treatment (Jeong et al., 2015). A collinearity diagnosis was performed for all factors. The VIF value of HBL was 25.00, and the VIF value of HBLI was 16.66, suggesting that HBL and HBLI are highly correlated with other independent variables. Therefore, sex, BMI, BGC, and MT were used for multiple linear regression analysis with the dependent variables. The specific results were shown in **Table 5**. A partial correlation analysis was performed between HBL, HBLI, and the dependent variables. The specific results were shown in **Table 6**.

**Table 4:** The results of the correlation analysis between risk factors and treatment outcomes.

	Age	Sex	BMI	MT	HBL	HBLI	BGC
CRP							
T1	0.03	-0.27*	0.36*	0.42*	0.70*	0.66*	0.76*
T2	-0.07	-0.35*	0.33*	0.56*	0.81*	0.76*	0.76*
T3	-0.14	-0.26	0.44*	0.29*	0.71*	0.64*	0.65*
T4	-0.21	-0.03	0.03	0.06	0.13	0.10	0.10
PCT							
T1	0.07	-0.26	0.20	0.37*	0.47*	0.43*	0.46*
T2	-0.15	-0.36*	0.20	0.55*	0.81*	0.69*	0.70*
T3	-0.29*	-0.20	0.03	0.17	0.27	0.23	0.23*
T4	-0.10	0.08	-0.10	0.04	-0.02	0.06	-0.09
ESR							
T1	-0.13	-0.24	0.54*	0.45*	0.79*	0.73*	0.76*
T2	-0.02	-0.29*	0.53*	0.61*	0.84*	0.79*	0.83*
T3	0.09	-0.29*	0.45*	0.67*	0.83*	0.76*	0.80*
T4	0.08	-0.42*	0.29*	0.44*	0.70*	0.54*	0.61*
WBCs							
T1	0.05	-0.12	0.22*	0.41*	0.41*	0.42*	0.40*
T2	-0.15	-0.14	0.22	0.40*	0.53*	0.50*	0.48*
T3	-0.12	-0.20	0.34*	0.35*	0.59*	0.48*	0.47*
T4	-0.02	-0.02	0.16	0.38*	0.31	0.31*	0.24*
VAS							
T1	-0.02	-0.09	0.34*	0.14	0.23	0.24	0.22
T2	0.04	-0.19	0.29	0.14	0.28	0.29*	0.19*
T3	0.14	0.02	0.03	0.23	0.28	0.33*	0.24*
T4	-0.00	0.17	-0.19	0.03	-0.14	-0.15	-0.11
DAT	-0.17	-0.42*	0.29*	0.55*	0.80*	0.74*	0.72*

*P value < 0.05.

**Table 5:** Multiple linear regression analysis results.

	Coefficient (B, 95% CI)	Standardized Coefficient (Beta)	P-value
T2CRP (mg/L)			
Sex	-5.63 (-13.62-2.37)	-0.13	0.16
BMI	0.70 (-0.94-2.33)	0.09	0.40
BGC	13.59 (6.29-20.90)	0.51	0.00*
MT	15.67 (1.19-30.14)	0.25	0.03*
T3CRP (mg/L)			
Sex	-3.93 (-14.33-6.48)	-0.08	0.45
BMI	1.24 (-0.89-3.37)	0.14	0.25
BGC	20.49 (10.98-29.99)	0.65	0.00*
MT	-6.58 (-25.42-12.27)	-0.09	0.49
T2PCT (ng/ml)			
Sex	-0.05 (-0.11-0.01)	-0.16	0.11
BMI	-0.01 (-0.02-0.01)	-0.09	0.43
BGC	0.12 (0.07-0.18)	0.60	0.00*
MT	0.09 (-0.02-0.20)	0.18	0.12
DAT (days)			
Sex	-4.7 (-8.36- -1.05)	-0.25	0.01*
BMI	0.31 (-4.37-1.06)	0.09	0.41
BGC	4.89 (1.55-8.23)	0.41	0.01*
MT	7.68 (1.07-14.30)	0.28	0.02*

*P value < 0.05.

**Table 6:** Partial correlation analysis results.

	Partial Correlation (R)	P-value
HBL (ml)		
T2CRP	0.42	0.00*
T3CRP	0.36	0.01*
T2PCT	0.49	0.00*
DAT	0.48	0.00*
HBLI		
T2CRP	0.40	0.03*
T3CRP	0.27	0.05
T2PCT	0.28	0.04*
DAT	0.50	0.00*

*P value < 0.05.

The results showed that BGC was strongly correlated with the severity of infection (Beta = 0.60, P = 0.00), strongly correlated with short-term treatment effectiveness (Beta = 0.65, P = 0.00), and moderately correlated with the DAT (Beta = 0.41, P = 0.01). HBL was moderately correlated with the severity of infection (Partial-R = 0.49, P = 0.00) and moderately correlated with the DAT (Partial-R = 0.48, P = 0.00). HBLI was moderately correlated with the DAT (Partial-R = 0.50, P = 0.00). Female was a favorable factor to shorten the DAT (Beta = -0.25, P = 0.01), and higher MT during infection may indicate a longer DAT (Beta = 0.28, P = 0.02).

## 4 Discussion

Although previous studies have generally confirmed that PED is a safe, effective, and minimally invasive procedure with a low incidence of complications, SSI in PED remain inevitable. Regarding the incidence of infection, Ogihara’s study indicated that the infection rate in PED is approximately one-third of that in traditional open surgery (Ogihara et al., 2018). Similarly, Hussein’s research reported comparable results (Hussein et al., 2014). According to Watanabe’s findings, continuous irrigation during PED may help reduce bacterial colonization at the surgical site, thereby lowering the risk of infection (Watanabe et al., 2010). Owen’s study also suggested that smaller surgical incisions are associated with a reduced probability of SSI (Owens and Wenke, 2007).

Risk factors for spinal postoperative infections include advanced age, male gender, obesity, a history of lumbar spine surgery, malnutrition, diabetes, and long-term corticosteroid use. However, some studies have indicated that higher BMI may be associated with a lower incidence of PED-related infections and earlier relief of postoperative VAS scores (Leyendecker et al., 2023). Nevertheless, the majority of studies suggested that obese patients are at higher risk for SSI, venous thromboembolism, longer operation times, and greater intraoperative blood loss.

Our analysis showed a weak positive correlation between BMI and the DAT (R = 0.29, P < 0.05), suggesting that obesity may not play a predominant role in culture-negative postoperative infections. The increased subcutaneous fat in obese patients likely promotes the release of inflammatory cytokines, leading to insulin resistance and, consequently, elevated blood glucose levels, which may increase the risk of infection exacerbation. Thus, BMI did not show a strong positive correlation with the DAT. In contrast, our study demonstrated a strong correlation between BGC and the DAT (R = 0.72, P < 0.05), likely due to the fact that elevated blood glucose levels persistently increase the risk of infection exacerbation, prolonging the need for antibiotic treatment. It should be noted that blood glucose levels may not directly affect the efficacy of antibiotics in treating infections, a relationship that warrants further investigation in future studies.

The cause of postoperative infections following PED remains unclear. However, two main theories are widely accepted in clinical practice. The first suggests that existing bacterial spread, such as from skin abscesses, endocarditis, or pharyngitis, could be the source of the infection. The second theory proposes that invasive procedures, such as surgery, trauma, or lumbar puncture, may directly introduce bacteria into the infection site (Naderi et al., 2003). The prophylactic use of antibiotics is currently the primary reason for preventing culture-negative infections. Given spinal infections destroy lumbar, nerve roots and the dural sac, SSI in the spine often presents with significant localized pain and radiating lower limb nerve pain (Tyrrell et al., 1999). Therefore, pain is often one of the earliest clinical signs of spinal postoperative infection. Whether localized to the surgical site or radiating along nerve pathways, pain should be given careful attention. However, like sterile inflammation, the early stages of infection may present with subtle systemic symptoms, causing localized pain to be mistakenly diagnosed as normal postoperative neuropathic pain. Since culture-negative infections prevent targeted antibiotic treatment, broad-spectrum antibiotics are often used empirically. Additionally, the presence of postoperative fever further complicates the accurate diagnosis of culture-negative infections (Dai et al., 2024). Therefore, the aim of this study was to conduct a comprehensive analysis of the clinical baseline characteristics, infection-related laboratory indicators, and imaging findings of patients, in order to identify the factors that significantly impacted the progression of anti-infection treatment. By addressing these risk factors, severe clinical infections could be prevented. The study hoped to provide valuable insights for the clinical management of patients with culture-negative infections following PED.

HBL, first identified by Pattison in 1973, is now defined as the extravasation of blood into tissue spaces and/or joint cavities, as well as the loss of hemoglobin due to hemolysis. Compared with VBL, HBL during PED accounts for the vast majority. In our study, HBL showed strong correlations with CRP, PCT, ESR, and VAS scores. This may be due to the fact that increased HBL leads to elevated local pressure, thereby intensifying pain symptoms. Accumulated HBL might also lead to local hemolysis, which could trigger the release of inflammatory mediators, resulting in neuroinflammation and intense neuropathic pain. Additionally, the accumulation of significant bleeding may result in the formation of local blood clots, which create an environment conducive to the buildup of inflammatory factors and bacterial colonization. Therefore, HBL serves as a significant risk factor for early increases in CRP, PCT, and ESR. In the later stages of infection treatment, as antibiotics take effect and local HBL is absorbed, HBL is no longer a major risk factor influencing the outcome of anti-infection therapy. Our findings supported these inference. Hence, controlling HBL is crucial for mitigating early infection progression. Previous studies have indicated that factors such as anesthesia methods, intraoperative medications, perioperative anticoagulants, blood pressure regulation during surgery, and even gastrointestinal ulcers can influence the volume of HBL (Guo et al., 2018; Xu et al., 2019). To ensure data accuracy, we introduced the HBLI. HBL and HBLI were similar in many characteristics. It further confirmed the certainty of the influence of HBL management during operation on the progress of postoperative infection.

In addition, our study found that gender is also a factor that affects the severity of infection and the therapeutic effect (Beta = -0.25, P < 0.05). Sex is a classified variable, and it is converted into a dummy variable, which is defined as “male = 0, female = 1” for the convenience of model calculation. Under this coding mode, the gender regression coefficient in the model indicates the influence of the mean difference between men and women on the dependent variable. Specific to the results of this study, women were the protective factors after infection, which was the same as the previous research conclusions (Ogihara et al., 2018).

Limitations: This study has several limitations: 1. As a retrospective study, the evidence is relatively weak, and prospective studies are needed to confirm the reliability of the conclusions; 2. Due to the nature of this study, it was not possible to establish an appropriate control group. Patients with negative cultures and those receiving targeted antibiotic treatment for positive cultures, as well as patients with aseptic inflammation who do not require antibiotics, could not form a convincing control group; 3. One limitation of our study was the lack of a suitable reference standard for grading BGC, which was instead based on clinical experience. This reliance on subjective grading may have reduced the robustness and generalizability of our findings. Further studies were needed to validate our conclusions; 4. Additionally, we did not account for the patients’ primary infectious conditions, such as chronic pneumonia and rhinitis, etc., which could have potentially influenced the effectiveness of the treatment and introduced bias into our results. Future research with larger sample sizes and multi-centre data analysis is needed to strengthen the validity of the study’s methodology.

## 5 Conclusion

To the best of our knowledge, this is the first study in the field of spine surgery to analyze the risk factors of antibiotic treatment in patients with culture-negative infections following endoscopic surgery. Our findings suggest that healthy blood glucose levels, a lower HBL and HBLI might help reduce the duration of antibiotic use after infection. Effective hemostasis during surgery to reduce HBL and good preoperative BGC indicators are both beneficial measures for infection treatment.

## Data Availability

The raw data supporting the conclusions of this article will be made available by the authors, without undue reservation.
